# Prostate cancer cells show elevated urokinase receptor in a mouse model of metastasis

**DOI:** 10.1186/1475-2867-6-21

**Published:** 2006-08-23

**Authors:** Inder Sehgal, Timothy P Foster, Joseph Francis

**Affiliations:** 1 LSU Department of Comparative Biomedical Sciences, Louisiana State University, Baton Rouge, LA 70803, USA; 2 LSU Department of Pathobiological Sciences, Louisiana State University, Baton Rouge, LA 70803, USA

## Abstract

**Background:**

The urokinase receptor (uPAR) governs several functions necessary during invasion and metastasis such as motility, degradation of the extracellular matrix and adhesion. This receptor has been recently associated with clinical prostate cancer progression. Experimentally, inhibition of uPAR reduces colonization of extra-prostatic sites in animal models. Our objective in this study was to compare uPAR expression in orthotopic vs. metastatic foci *in vivo *and to examine at the cellular level how uPAR might promote early stages of metastasis.

**Results:**

We show that uPAR staining is significantly greater in regional lymph node metastases than in the intraprostatic tumor mass. Using transient over-expression, we found that uPAR increases *in vitro *motility and chemotactic invasion. Finally, we demonstrate that uPAR is up-regulated by a significant subpopulation prostate cancer cells following matrix detachment and maintenance in suspension and we provide evidence that prostate cancer cells with elevations in uPAR have an enhanced resistance to anoikis.

**Conclusion:**

These data provide new evidence that uPAR can be induced by cancer cells during metastasis in vivo and that this elevated uPAR enhances resistance to anoikis in vitro.

## Background

Successful metastasis entails acquisition of phenotypic qualities that may be atypical to the majority of cells within the primary tumor mass [1]. These traits include enhanced motility, increased degradation of the extracellular matrix, altered adhesion and the ability to survive apart from the homeotypic environment [2]. Although large numbers of tumor cells begin the invasive process, those cell numbers are greatly reduced through selection for the ability to survive or grow through the sequences of metastatic progression [3,4]. Much remains to be learned about specific mechanisms which permit regional metastasis followed by dissemination to distant sites; however these mechanisms are generally understood to incorporate responses to exogenous growth factors and cytokines, regulation of matrix proteases, interaction with stromal elements in the tumor microenvironment, angiogenesis, resistance to apoptosis and avoidance of immune surveillance [5,6].

uPAR regulates cancer dissemination in a variety of ways. By binding to its enzymatic ligand uPA which generates plasmin, uPAR directs extracellular matrix (ECM) degradation necessary for cancer cell invasion. By placing uPAR at the leading edge of directional migration, cells are able to limit the scope of this matrix proteolysis. uPAR also interacts with the ECM through its binding to vitronectin and associations with various integrins and other extracellular proteins. These associations allow uPAR to use the ECM as a scaffold to support cell motility, spreading and adhesion during tumor invasion [7]. uPAR also generates discrete signaling cascades most likely through contact with transmembrane proteins. These internal signals have been reported to induce growth, actin cytoskeletal rearrangements, and protection from apoptosis [7-9]. Recently, uPAR overexpression was shown to inhibit anoikis in non-transformed human retinal pigment epithelial cells. This effect resulted from MAPK and phosphatidylinositol 3-kinase (PI3K) upregulation of the Bcl-xL anti-apoptotic factor [10].

With regard to human prostate cancer, elevations in a soluble form of plasma uPAR is associated with tumor development, progression and worse prognosis [11-13]. Tumor-associated uPA and uPAR have been reported to be preferentially expressed in prostate adenocarcinoma cells vs. prostatic stroma [14]; however, this cancer cell expression is not consistently found in patient samples [15] suggesting that uPAR expression in primary tumor tissue may be very low or negative in some instances. The prognostic significance of uPAR expression in prostate cancer metastases such as bone has not been reported; however human gastric cancer patients with elevated uPAR expression on tumor cells which disseminated to bone marrow have shorter disease-free and overall survival [16].

In animal models of prostate cancer, pharmacological inhibition of uPAR reduced bone colonization of PC3 cells when the cells were administered directly into the vasculature [17] and lung, liver and lymph node metastases are reduced by anti-uPAR antibody treatments in a subcutaneous rat prostate model [18]. Pulukuri et al. [19] have recently demonstrated that shRNAs for uPA and uPAR almost completely inhibit established orthotopic tumor growth; however, little is known about the expression of uPAR during prostate cancer progression from the orthotopic environment to early metastatic sites.

In this report, we sought to determine if a relationship exists between uPAR expression and development of early metastatic foci *in vivo*. Next, we addressed at the cellular level, how uPAR overexpression in a relatively small fraction of prostate cancer cells might promote invasion and metastasis. We found that transient overexpression of uPAR promotes selective increases in both *in vitro *motility and chemotactic invasion. In addition, we link for the first time uPAR with the ability of tumor cells to resist anikois – the induction of apoptosis which follows the loss of adhesion. Our study indicates that uPAR may be temporarily overexpressed in a sub-fraction of tumor cells permitting those cells to survive the initial lethal first stages of metastastic progression.

## Results

### PC-3M tumors metastasize to regional lymph nodes and express increased uPAR

**Figure 1:**
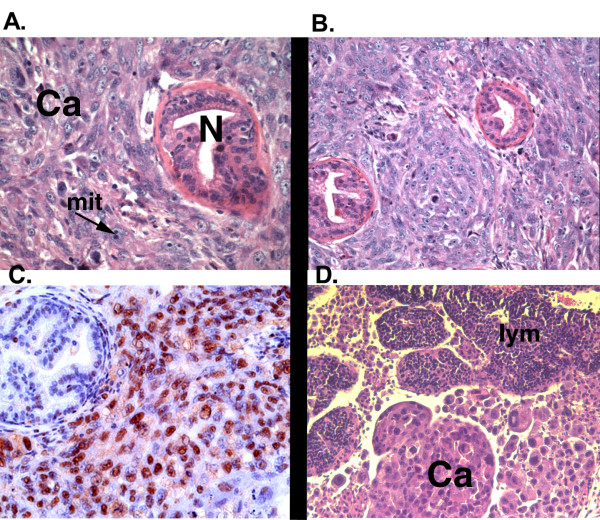
PC-3M prostate cancer and metastasis. PC3M-luc cells (5 × 10^5^) were orthotopically injected into the prostate of Nu/Nu mice. Mice sacrificed at 50 days, prostate tumors were removed and prepared for sectioning. A. H&E section of orthotopic tumor [400×] mass. Normal glands (N), cancer cells (Ca) and mitotic figure (mit) are indicated. B. H&E section of tumor [200×] compressing normal glands. C. PCNA staining of PC-3M orthotopic tumor [630×]. D. Lymph node metastasis [200×] showing cancer cells (Ca) and lymphatic tissue (lym).

Histologically, PC-3M tumors which developed in the prostates of nude mice exhibited pleiomorphic morphology and a high mitotic index (Figure 1A). The primary tumor often compressed existing mouse prostatic acini (Figure 1B). These orthotopic tumors exhibit robust expression of PCNA indicative of active proliferation (Figure 1C). Each mouse also developed several metastases to regional lymph nodes in proximity to the abdominal aorta. Tumor cells within these lymph nodes developed discrete foci which were surrounded by lymphatic tissues (Figure 1D).

**Figure 2:**
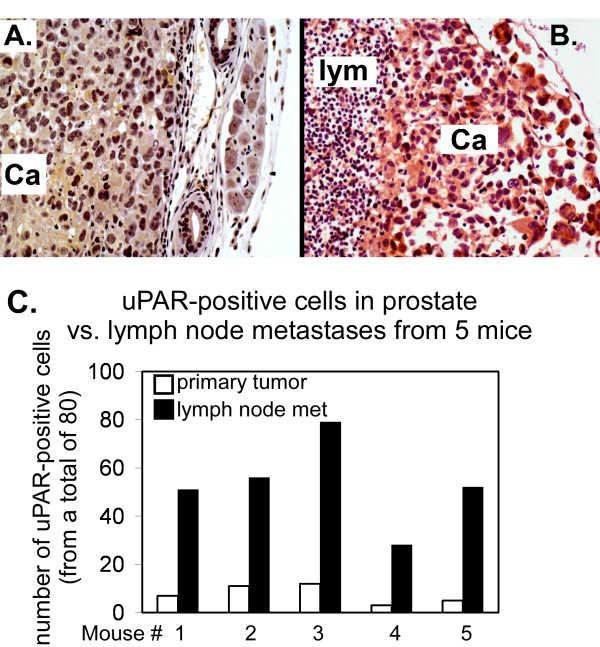
uPAR-positive cells in prostate cancer vs. lymph node metastases (200× images). Tumor section images taken from one representative mouse and stained with rabbit anti-uPAR (399R, American Diagnostica Corp.). A. Little or no uPAR staining is apparent in the cancer (Ca) cells within the mouse prostate. Image taken at tumor periphery. B. Strong uPAR immunoreactivity in metastatic cancer cells (Ca) is seen adjacent to lymphatic cells (lym).C. uPAR-positive cells in prostate vs. lymph node (l.n.) metastases in each of 5 mice. Data show the total number of uPAR-positive cells counted in each prostate and l.n. for each mouse. White bars represent primary tumors, black bars represent the l.n. metastases. The data for all 5 mice was collectively analyzed using a paired t-test and the p value was 0.0011.

The primary tumor and tumor-bearing lymph nodes from the abdominal region were removed and immunochemically stained for uPAR. There was little uPAR-positive staining in the primary tumor mass, indicating that high uPAR expression is not requisite for establishment or proliferation of the primary tumor in this model (Figure 2A). Staining of tumor cells proliferating within regional lymph nodes however, revealed multifocal regions possessing deep staining indicative of uPAR positivity (Figure 2B). Often these metastatic epithelial cells were more heterogeneous in size than those in the primary tumor. To quantitatively compare uPAR expression between primary and lymph node tumors, tissue sections were randomly scored to determine the number of uPAR-positive cells. These data confirmed a highly significant increase in uPAR positive cells in lymph node metastases compared with uPAR positive cells in the primary tumors (Figure 2C) in each of the mice.

### uPAR confers increased migration and invasive properties to transfected PC-3M cells

In order to begin to understand why uPAR might be enriched in lymph node metastases, we directly compared uPAR overexpressing (uPAR+) cells with wild-type cells *in vitro *in assays designed to mimic some of the processes required for successful invasion We introduced uPAR into PC-3M cells by transient tranfection; this method produced a fraction of uPAR+ cells accompanied by a majority of wild-type uPAR-expressing PC-3M, which was analogous to our observation in the orthotopic tumor.

To measure migration, cells were applied on top of FluoroBlok transwell chambers. The percentage of uPAR+ cells which traversed the insert was compared with the percent of uPAR+ cells remaining in the upper chamber (Table 1) and results indicated that the enrichment of uPAR+ cells in the migrating fraction was significant. In addition to migration, the ability to degrade and invade through extracellular matrix is a key component in successful early metastasis. Therefore, we next coated the FluoroBlok membranes with Matrigel and repeated the assay. We found that transient uPAR over-expression increased PC3M invasiveness as the population of invading cells possessed a much greater percent of uPAR+ cells (Table 1).

**Table 1:** uPAR overexpression enhances directional migration and *in vitro *invasion of PC-3M cells.

		migration	invasion
uPAR transfected cells	upper insert	15.0 ± 3.0%	20.0 ± 2.0%
	lower insert	29.4 ± 4.9%	55.0 ± 12.0%
β-gal plasmid transfected (control) cells	upper insert	18.4 ± 3.2%	14.5 ± 2.8%
	lower insert	16.1 ± 1.9%	12.0 ± 1.5%

Table data indicate the percent uPAR+ or β-galactosidase+ cells in the population of cells on the upper membrane (general cell population) and lower membrane (cells which successfully traversed the membrane barrier) after a two day period. Data shows the mean and S.E.M of 3 replicate experiments.

In order to determine if transfection itself lead to altered motility and/or invasion, PC-3M cells expressing EGFP were transfected using a control plasmid (pEF6/V5 His lacZ). We found no differences between the percents of β-gal-immunoreactive transfected cells above or beneath the transwell membranes indicating that the transfection procedure per se did not account for the observed increase in motility and invasion (Table 1).

### uPAR cell surface expression in induced by culture in suspension and is associated with reduced levels of anoikis

**Figure 3:**
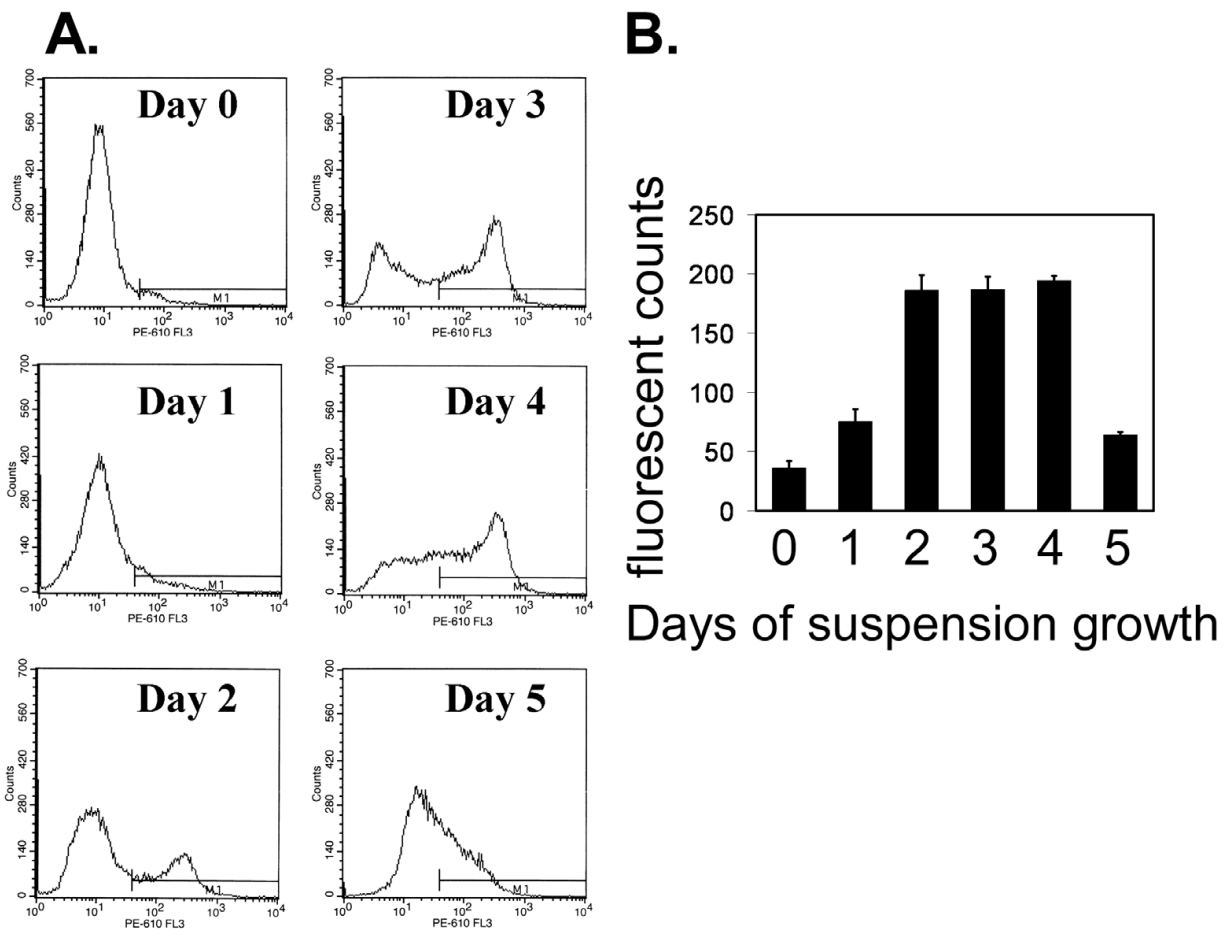
Flow cytometry of uPAR cell surface expression shows development of a high uPAR population during suspension culture. A. Representative flow cytometry histograms of uPAR expression on PC-3M cells over a 5 day time course in forced suspension culture. M1 marker shows region greater than background fluorescence (secondary antibody-only control). B. Bars represent the mean fluorescent intensity measured in fluorescent counts of uPAR expression in un-fixed PC-3M cells. Cells were incubated in suspension as described in methods for the time periods shown. Bars are the mean of 4 replicates ± s.d.

One critical barrier to successful metastasis is survival during the period of matrix detachment. This detached state can be modeled *in vitro *by a forced culture in suspension [4]. PC-3M cells were incubated in suspension over a period of 5 days and then assessed by flow cytometry for uPAR cell surface levels. This experiment demonstrated a division of the overall cell population with the development of a sub-population substantially shifted towards an increase in uPAR. This shift began after only one day of suspension (Figure 3A&B). This uPAR-induced sub-population peak continued to rise through the second day and then plateaued until sharply declining after 5 days of culture. These data indicated that uPAR was induced during a period of matrix deprivation and suggested the possibility that this induction might be associated with cell survival or death.

**Figure 4:**
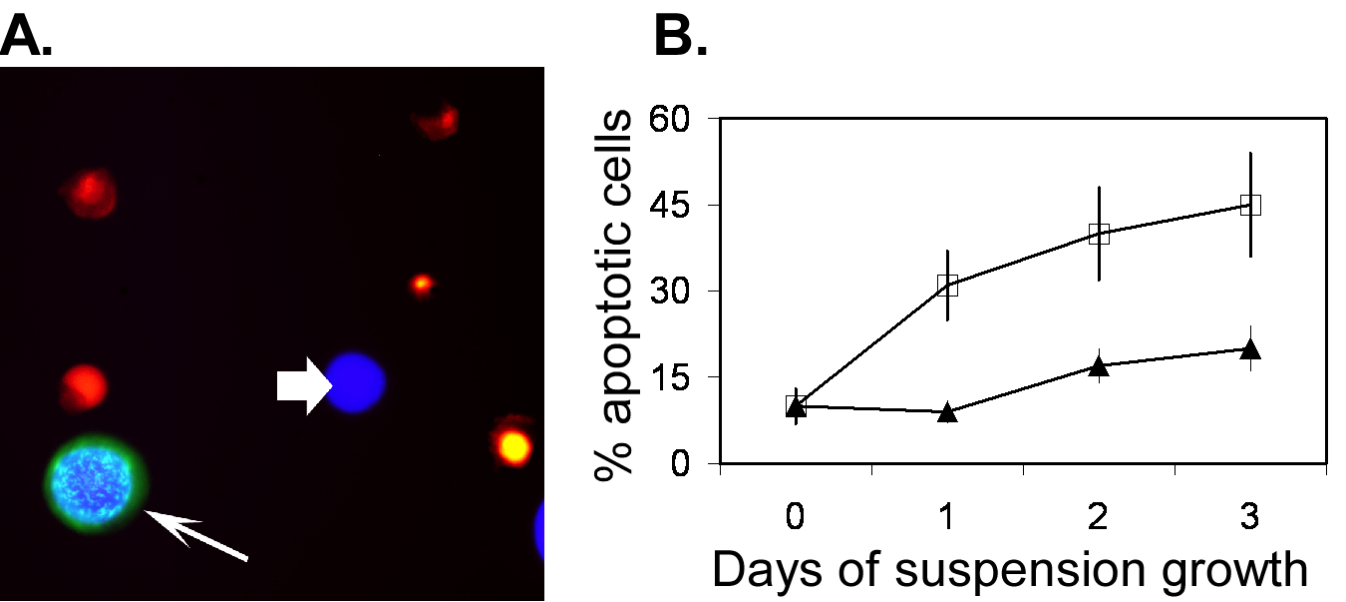
Immunohistochemical staining and quantitation of anoikis in PC-3M cells. A. uPAR over-expression and apoptosis in PC-3M suspension culture. Thin arrow indicates a fluorescence overlay of a live cell (blue) which over-expresses uPAR (green). The cell surface uPAR expression is characterized by a stippled staining pattern in the central region of the cell and forms a peripheral hallow. This uPAR+ cell is easily distinguishable from a live cell with wild type-uPAR expression (wide arrow). Apoptotic cells are shown with nuclear up take of orange-red staining from ethidium bromide. The nuclei demonstrate various degrees of condensation while the cell body show no up take of Calcein blue. B. The percentage of apoptosis in cells with wild-type (□) and uPAR+ (▲) are plotted over a 3 day period of suspension. Apoptosis in individual cells was defined as cells which allowed ethidium bromide to permeate and stain the nucleus (indicative of a breached plasma membrane) and were unable to accumulate Calcein blue methoxyester (fluorescent marker for live cells). uPAR over-expressing cells were identified by immunofluorescent staining using Alexa Fluor 488. Data represent mean ± S.D. of four replicate samples.

To pursue this possibility, we investigated the levels of anoikis in uPAR+ and wild-type PC-3M cells. uPAR-transfected populations of PC-3M were cultured in suspension over a 3 day period. The time course was limited to the 3 day period to ensure adequate transient expression of the uPAR protein. Aliquots of cells were removed daily and scored for number of live, apoptotic and uPAR-over expressing cells (depicted in Figure 4A). Data from this experiment revealed that uPAR conferred a protective effect against developing anikois during 3 days of suspension incubation (Figure 4B).

## Discussion

We have used an *in vivo *assay of orthotropic metastasis to regional lymph nodes to begin to dissect the role of uPAR during early stages of prostate cancer invasion and metastases. This model demonstrated that metastases which succeed in invading as far as these lymph nodes and then proliferated within the nodes possessed significantly greater numbers of uPAR-positive staining cells when compared to the general population of cells within the primary tumor. This finding is in accord with a recent report using cDNA microarray of PC-3M cells to identify genes upregulated in lymphatic metastasis [20]. The apparent up-regulation of uPAR in lymph node tumor colonies suggests that uPAR expression is a phenotypic trait that is positively selected for during metastasis. While increased expression could result from the expansion of a few cells from the primary tumor which over-expressed the receptor, the increase could also be an inducible phenomenon by which cells expressing little or no uPAR in the primary tumor induce the uPAR within metastatic environments. Our *in vitro *data supports this second possibility for cancer cells in environments without matrix attachment as we found some suspended PC-3M cells gradually up-regulated surface uPAR expression.

In order to identify specific traits that elevated uPAR could impart to prostate cancer cells which colonized regional lymph nodes, we transiently transfected uPAR into PC-3M cells and examined cell behavior *in vitro*. uPAR-over expression in a fraction of PC-3M cells conferred increased directional motility as well as increased invasion. These increases match well with the biology of uPAR action since uPAR binds to vitronectin and specific beta integrins allowing it to mediate motility. uPAR also binds to its enzymatic ligand uPA, which can initiate a cascade of protease activities which degrade most non-collagenous components of the extracellular matrix [8].

In addition to assessing behaviors necessary to gain access to the lymphatic vessels, we next considered the possibility the uPAR might help tumor cells survive within these vessels. Recent evidence indicates that epithelial cancers have a high mortality within the circulation [3] due in large measure to a form of apoptosis induced by matrix detachment and existence in suspension within the blood or lymphatic vessels. This form of apoptosis is termed "anoikis" [21]. We examined the effect of suspension culture on uPAR cell surface expression by flow cytometry. Through this experiment, we documented that uPAR is induced in a distinct subpopulation of the suspended tumor cells giving rise to two peaks – one of low constitutive uPAR levels and one of uPAR-induction. These two populations remained until the fifth day of growth in suspension. This novel association of uPAR with culture in suspension and the well-known link between suspension and apoptosis, promoted us to next examine the effect of uPAR overexpression on anoikis resistance. We discovered that uPAR+ cells were less likely to undergo late-stage apoptosis over a 3 day period than wild-type PC-3M. Such a delay in anoikis conferred by uPAR could allow prostate cancer cells to survive in hematogenous or lymphatic circulation while they migrate to a secondary site. A recent report by Alfano et al [10] using nontransformed retinal epithelial cells determined that overexpression of uPAR can induce resistance to apoptosis including anoikis. Our data extend this observation to PC-3M prostate cancer cells and further demonstrates that these cancer cells have the ability to increase uPAR during suspension. It will now be important to determine if matrix deprivation leads to increased uPAR expression in other prostate and other types of cancer lines and if increased uPAR is accompanied by a complementing increase in uPA.

Although the wild-type PC-3M cell line expresses a moderate level of uPAR – higher levels than PC-3 and lower levels than PC-3MM2 cells [22] – these cells still under went a significant degree of apoptosis indicating that the basal uPAR expression does not confer the degree of resistance offered by over-expression of uPAR. In this report, we have not attempted to study any anti-apoptotic efficacy of this basal uPAR expression. Although lowering basal uPAR could lead to greater aniokis; we believe that this type of experiment would be more useful in a population of cells with a high percentage of uPAR-overexpression and we are currently working towards this goal using a viral delivery system.

The mechanism through which uPAR expression delays the onset of anoikis in cancer cells must be elucidated. uPAR-induced resistance to anoikis in the nontransformed retinal epithelial cells resulted from uPA binding and activation of MEK/ERK and PI3K/Akt pathways. The mechanism in prostate cancer cells could originate with two other protein families, integrins and caveolin, which have been shown to confer resistance to anoikis in these cells. Integrins appear to activate a focal adhesion kinase (FAK)/ phosphoinositide 3-kinase (PI3K)/AKT pathway [21,23] while caveolin-1 both activates Akt and inhibits Akt inhibitors [24,25]. Suppression of anoikis can also result through inhibition of the Jun amino-terminal kinase (JNK) apoptotic pathway by ERK activation [24]. Since uPAR can transduce signals which activate ERK in MCF-7 breast cancer cells [26], perhaps a similar ERK activation pathway exists in prostate cancer.

## Conclusion

Overall, our *in vivo *model indicates that there is a selection for prostate cancer cells with high uPAR expression during early stages of successful metastasis. *In vitro*, high uPAR expression enables prostate tumor cells to become more motile and to behave more invasively. It also may provide a temporary inhibition of the highly lethal apoptotic mechanisms which are triggered during matrix-free periods that the cancer cell must transition through moving from primary to metastatic sites. Our finding that uPAR is induced in some cells during forced-suspension culture and that uPAR+ cells are more resistant to anoikis is particularly relevant because few other cell surface proteins have been linked to resisting suspension-induced anoikis. Further dissection of uPAR functioning during the metastatic cascade will provide greater insight into why this receptor is upregulated in regional metastasis.

## Methods

### Cell culture

PC3M cells (a gracious gift from Dr. Isiah Fidler, MD Anderson Cancer Center, Houston TX) were maintained in Minimal Essential Medium with Earle's salts (MEM), supplemented with 10% fetal bovine serum, 100 units/mL penicillin, 100 μg/ml streptomycin, and 2.0 mM L-glutamine.

### Intraprostatic tumors

For orthotopic tumor inoculation, 8–10 week old Nu/Nu male mice (Charles River labs) were anesthetized with isoflurane. A low abdominal skin incision cranial to the prepucial glands was made and the seminal vesicles were carefully exteriorized to expose the dorso-lateral prostate. Using a 29 gauge insulin syringe, 5 × 10^5 ^PC-3M cells suspended in MEM were injected into the dorso-lateral prostate in a 20 uL volume. The seminal vesicles and prostate were held for one minute to allow the injected cells to settle into the gland and then gently replaced into the abdominal cavity. Body wall and skin wounds closed were closed with 5-0 PDS and 5-0 nylon respectively. All animal procedures were approved by the LSU IACUC committee.

Tumors were allowed to develop for 50 days. The primary tumor was removed during necropsy and fixed in formalin then paraffin embedded, sectioned and stained with H&E. Enlarged lymph nodes from the paralumbar region were visualized under surgical microscopy and then dissected out. These lymph nodes were then fixed, embedded and histologically analyzed for prostate cancer cells.

### Tissue immunostaining

Formalin-fixed prostate tumor tissues were paraffin embedded, sectioned, applied to Plus slides (VWR Corp) and then stained using a Dako autostainer system. The slides were pre-treated with 3.0% hydrogen peroxide for 10 minutes, then rinsed and treated with a 10 μg/mL solution of proteinase K solution for 3 minutes to enhance antigen retrieval. Next, non-specific binding sites were blocked by addition of normal goat serum for 30 minutes and then a 10 μg/mL solution of rabbit anti-human uPAR (antibody 399R, American Diagnostica) or rabbit anti-human Proliferating Cell Nuclear Antigen (AB15497, AbCam antibodies) was applied to the tissue for 30 minutes. Primary antibody was removed by washing and then appropriate horseradish peroxidase-labeled secondary antibody was applied for a 30 minute period and was subsequently detected using NovaRed substrate (Vector Labs, Burlingame, CA) in a 8 minute incubation. Slides were briefly counter stained using hematoxylin before drying.

Cells from slides of primary and lymph node sections were scored as either positive or negative for uPAR. Four regions of each slide were randomly selected and 20 cells from each region were scored. Data was collected from 5 different mice and uPAR staining in tumors was compared to lymph node metastases from the same mouse.

### Plasmids constructs and transfection

The 1.0 kb coding region of human uPAR was amplified using PCR from a pT7TSuPAR plasmid [[27], a gift from Dr. K. Karico, U. Pennsylvania] then cloned into the pEF6/V5 His TOPO plasmid using the pEF6/V5 His TOPO kit (Invitrogen Corp). This plasmid transcribes target cDNA using the EF-alpha promoter.

Cells were transfected using a liposome (Lipofectamine 2000, Invitrogen). PC-3M cells were transiently transfected with 1.0 μg per 24-well of uPAR plasmid using 1.0 μL liposome. Transfection medium was changed after 8 hours. Approximately 15% of cells over-expressed uPAR after a 2 day period using this procedure as determined by immunofluorescence.

### Two chamber migration and *in vitro *invasion assays

Using a PC-3M cell strain that we have previously transfected and selected to express EGFP driven by the EF-alpha promoter, the uPAR cDNA was transiently introduced into the cells using liposomal transfection. Over-expression of uPAR was verified using immunostaining and fluorescent microscopy. Cells were then plated into the upper chamber of 24-well compatible Fluro-Blok transwell inserts (BD Falcon) using 200,000 cells per insert. For invasion assays, these inserts were pre-coated with 25.0 μg per membrane of Matrigel (BD Falcon). Cells placed on top of the Matrigel layer must degrade the extracellular matrix to reach the transwell porous membrane and then migrate through the pores to the chemoattractant. Migration assays utilized uncoated transwells.

The inserts were placed into standard culture medium supplemented with 25.0% fetal bovine serum to serve as a chemoattractant. The bottom of the membrane was visually inspected after 2 days for appearance of the EGFP-expressing cells indicative of cells traversing the membrane. In order to quantitate the numbers of uPAR-over expressing cells as a ratio of the total cells, the following methodology was used. After a 2 day period, three inserts were immunostained for uPAR by sequential submersion in 0.4% paraformaldehyde fixation solution × 20 minutes, 0.1% Triton-X 100/PBS permeabilization solution × 3 minutes, HBSS/2% goat serum blocking solution × 10 minutes, blocking solution with 2 μg/mL mouse anti-human uPAR (MAB 807, R&D Systems) × 2 hours, HBSS wash solution, blocking solution with 2 μL/mL Alexa Fluor 595 goat anti-mouse secondary antibody × 1 hour, PBS as a wash solution. The membrane in the transwell was carefully removed from the transwell, inserted between two coverslips and both the upper and lower sides were viewed under epifluorescence. The Fluro Blok membrane effectively blocks fluorescence from cells on one side of the membrane from appearing on the other side. Using the 20X objective, five fields of view were randomly scored for cells showing uPAR-over expression (seen as red cells) and total cells (seen as green).

### Flow cytometry assay to assess uPAR induction during suspension culture

PC-3M cells from stock adherent cultures were removed from surfaces with 0.4% EDTA in PBS, washed to remove EDTA and resuspended in growth medium. Approximately 10^6 ^cells per tube were placed in 50-mL polypropylene conical tubes to initiate apoptosis in suspension [4]. Four sets of tubes were prepared each day for periods of 5 days after which the cells and a freshly prepared control group (day 0) were prepared for flow cytometry. The cells were pelleted, re-suspended in Hank's Buffered Saline solution (HBSS) containing 2% goat serum and then incubated with 2 μg/mL mouse anti-human uPAR (R&D Systems) for 2 hours at 37°C. Cells were then washed in HBSS and incubated with Alexa Fluor 610 – R-phycoerythrin goat anti – mouse IgG conjugate secondary antibody for one hour. The mean fluorescent intensity (MFI) of uPAR expression on cells gated above background (M1) was calculated for the samples over the 5 day time course.

### Assay to determine anoikis

PC-3M cells were transiently transfected overnight. To initiate apoptosis as a result of detachment (anoikis), cells were removed from culture surfaces with 0.4% EDTA in PBS, washed to remove EDTA and resuspended in growth medium supplemented with heat-inactivated FBS. This cell suspension was placed in a 50-mL polypropylene conical tube to prevent re-attachment [4] with a perforated cap to allow air passage. Aliquots of the cell suspension were analyzed each day over 3 days to determine rates of anoikis.

A modification of a commercially available live-dead assay system (Molecular Probes) was used to differentially stain apoptotic and live PC-3M cells. A one mL aliquot of cells was loaded with 10 μg/mL Calcein Blue AM (Molecular Probes) for 20 minutes at 37°C. This esterified probe is a marker for live cells as it penetrates cells with an intact outer membrane and is hydrolyzed by intracellular esterases freeing the fluorescent Calcein blue (excitation peak 373 nm, emission peak 440 nm). The cell solution was then treated with 1 ug/mL ethidium bromide, which readily penetrates ruptured cell membranes intensely staining the DNA. Ethidium staining of condensed nuclei is an indication of late-stage apoptosis. The ethidium fluorescent can be excited at 510 nm and detected at 595 nm. Cells were then briefly pelleted by centrifugation, the medium was removed and replaced with PBS and the pellet was re-suspended. Cell samples were then viewed using epifluorescence and at least 20 cells per objective field were scored as either 1) apoptotic and wild-type uPAR, 2) apoptotic and uPAR-over expressing, 3) live and wild-type uPAR or 4) live and uPAR-over expressing. Nine fields were scored for each sample. To identify cells with over-expressed uPAR in the cell solution, the aliquoted solution was incubated for 6 hours with 4 μg/mL mouse anti-uPAR (R&D Systems MAB 807), then for an additional hour with goat anti-mouse Alexa Fluor 488. This procedure resulted in the cell surface labeling of uPAR-over expressing cells with a green fluorescent dye easily distinguishable from the blue and orange spectra of the live-dead stains. The uPAR levels in wild-type PC-3M cells was very low compared with levels in transfected cells and these wild-type cells did not demonstrate visible stain.

### Immunofluorescence

Cells were fixed in ice cold paraformaldehyde for 10 minutes then permeabilized using a solution of 0.1% Triton X-100 in PBS for 5 minutes. Nonspecific primary antibody binding was reduced by first blocking the cells for 20 minutes using a solution of HBSS with 2% normal goat sera. Primary antibody (anti uPAR monoclonal, R&D Systems MAB 807) was then added for 1.5 hours, and after the cells were washed, a fluorescent-tagged secondary antibody (Alexa Fluor 488 or 594, Molecular Probes Inc.) was applied. Fluorescence was imaged through a Zeiss Axiovert 200 fluorescent microscope and recorded through an Olympus Q-Capture 5.1 mega pixel color digital camera.

## Abbreviations

uPAR urokinase receptor

uPAR+ cells uPAR overexpressing cells

uPA urokinase-type plasminogen activator enzyme

EGFP enhanced green fluorescent protein

PCNA proliferating cell nuclear antigen

MEM minimal essential medium

H&E Hematoxylin and eosin stain

HBSS Hank's balanced salt solution

MFI mean fluorescent intensity

## Competing interests

The author(s) declare that they have no competing interests.

## Authors' contributions

IS performed experimental procedures, study design and drafted the manuscript. TPF designed and constructed expression vectors and initiated the immunofluorescent technique. JF participated in the development and design of in vivo procedures and immunohistochemistry. All authors read and approved the final manuscript.
